# Cognitive impairment and associated factors among adults with type 2 diabetes mellitus in eastern Sudan: a cross-sectional study

**DOI:** 10.3389/fendo.2026.1876049

**Published:** 2026-08-18

**Authors:** Saeed M. Omar, Hatim Y. Alharbi, Afnan A. Alwabili, Sami S. Alharthi, Ishag Adam

**Affiliations:** 1 Department of Medcine, Faculty of Medicine and Health Sciences at the University of Gadarif, Gadarif, Sudan; 2 Department of Psychiatry, College of Medicine, Qassim University, Buraydah, Qassim, Saudi Arabia; 3 Department of Medicine, College of Medicine, Taif University, Taif, Saudi Arabia; 4 Department of Obstetrics and Gynecology, College of Medicine, Qassim University, Buraydah, Qassim, Saudi Arabia

**Keywords:** age, cognitive impairment, diabetes mellitus, education, male, Sudan

## Abstract

**Background:**

Cognitive impairment in patients with diabetes mellitus (DM) is one of the major global public health issues. However, data on this issue remain scarce in Sudan. This study aimed to investigate the prevalence of cognitive impairment and its associated factors among adults with type 2 DM (T2DM) in East Sudan.

**Methods:**

A hospital-based cross-sectional study was conducted at the Gadarif DM center. Using a systematic sampling method, 400 adults aged ≥18 years were recruited. Cognitive status was assessed using the Montreal Cognitive Assessment (MoCA), with a score of<26 indicating cognitive impairment. Multivariate binary analysis was performed.

**Results:**

Of 400 patients with T2DM, 276 (69.0%) were females. The median (interquartile range, IQR) of the age and duration of T2DM were 53.0 (45.0–61.0) years and 7.0 (2.1–12.0) years, respectively. Three hundred and six (76.5%) patients with T2DM had cognitive impairment.

Multivariate logistic regression showed that lower education level (OR = 9.93, 95.0% CI = 5.51–17.90), non-employee status (OR = 2.23, 95.0% CI = 1.16–4.29), and alcohol status (OR = 8.21, 95.0% CI = 1.02–66.02) were associated with cognitive impairment. There was no association between age, sex, marital status, residency, smoking status, hypertension, hyperlipidemia, BMI, hemoglobin A1c/poor control of T2DM, and cognitive impairment.

**Conclusion:**

Three out of four patients with T2DM had cognitive impairment in East Sudan. Having a low level of education, non-employee status, and alcohol consumption are the associated factors for cognitive impairment. There is a need for public health interventions so as to detect and manage cognitive impairment early in adults with T2DM.

## Introduction

Diabetes Mellitus (DM) is a group of metabolic diseases characterized by hyperglycemia, which eventually causes microvascular and macrovascular complications (1). As of 2022–2024, approximately 800 to 830 million adults worldwide are living with DM, with Type 2 diabetes mellitus (T2DM) constituting about 95–98% of these cases (2). Moreover, the global prevalence of DM among adults has doubled since 1990, rising from 7% to approximately 14%, with the highest rates in low- and middle-income countries (2). Several complications, such as neuropathy, nephropathy, retinopathy, cardiovascular disease, peripheral artery disease, diabetic foot ulcer, gum and mouth problems, and sexual problems, are common complications attributed to DM (3).

A high rate of cognitive impairment in patients with DM has been reported in sub-Saharan Africa (4–9). Moreover, several factors, such as older age (4, 6, 8, 9) a low level of education (4, 6, 7) rural residence (5), alcohol (5, 7), obesity (4, 8), fasting blood sugar (4, 5, 7), poor control of DM (6), duration of DM (6, 9), co-morbidity (5, 7) and hyperglycemic episodes (4) have been reported to be associated with cognitive impairment in patients with DM (4).

Diabetes mellitus in Sudan is a growing public health problem. The International Diabetes Federation estimated that 3.5 million adults had DM in 2021 and projected that this number will reach approximately 8.7 million in 2050 (10). Since April 2023, there has been an ongoing conflict in Sudan, which has led to the disruption of the health system. Patients with DM faced challenges accessing testing and medications, in addition to storing insulin because of frequent power outages, transportation, and long wait times or overcrowding. These factors have forced patients to adopt hazardous coping mechanisms (11). There are few published data on cognitive impairment in Sudan (12). There is a need for research on cognitive impairment in patients with DM so as to yield data that could be utilized for health planning and interventions. This study aimed to investigate the prevalence of cognitive impairment and its associated factors among adults with type 2 DM (T2DM) in East Sudan.

## Methods

### Study setting, design, and population

This cross-sectional study was conducted between October and November 2025 among patients attending the Gadarif Diabetes Center in Gadarif, Eastern Sudan, in accordance with the Strengthening the Reporting of Observational Studies in Epidemiology (STROBE) guidelines (13). This tertiary hospital is the leading adult diabetes follow-up center in Gadarif state, providing medical follow-up services, nutritional counseling, and diabetic wound care for affected patients. All patients with DM attending the follow-up clinic were eligible to participate in the study.

### Inclusion and exclusion criteria

Adult patients (≥18 years old) with T2DM who had been under follow-up for at least 4 months were included. The standard blood test for tracking diabetes control, HbA1c, reflects the average blood sugar over the previous 2 to 3 months. A 4-month follow-up ensures that enough time has passed to accurately evaluate the impact of interventions (such as strict glycemic control, insulin adjustments, or cognitive training) on both glycemic and cognitive outcomes. Those who were critically ill or who refused to participate in the study were excluded. Moreover, adults who had hearing loss were excluded from the study.

### Sample size calculation

The sample of 400 adults with T2DM was calculated using OpenEpi Menu (OpenEpi, Atlanta, GA, USA) (14), which computed the formula $n=\frac{Z^{2}p.\left(1-P\right)}{d^{2}}$. Where n = sample size, Z= confidence level (1.96), p = estimated prevalence, and d=Margin of error to be tolerated. Because there was no prior data on cognitive impairment among adults with T2DM in Sudan, a sample size of 400 participants was calculated. This was based on a prior study from Northwest Ethiopia where the prevalence was 28.3% (5). To detect a 5% difference (α=0.05, 80% power), we also used the previous study’s assumptions that males comprise 55.0% of the cognitively impaired cohort and 40.0% of the unimpaired cohort (5).

### Sampling technique

To prevent selection bias, a systematic sampling method was applied by enrolling every second patient. The sampling interval (*k*) was determined by dividing the population size—the 982 patients with T2DM who attended the center during the previous three months—by the calculated sample size of 400 (*k* = 982/400) ≈ 2.

### Data collection

The main author (SMO) delivered essential training to the medical officers (one male and one female data collector) on the data collection procedure and the questionnaire itself over the course of one day. Questionnaires were checked for completeness and accuracy. The relevant scale content was explained in detail to the respondents to ensure they understood it. Every day, the main author conducted a quality check at the center to eliminate any errors in the data collection process. A questionnaire was administered via face-to-face interviews. The questionnaire for this study was developed based on prior studies. It included the patients’ age (in years), sex (male or female), marital status, educational level (less than or equal to secondary level or more than secondary level), residence, and occupation (employed or non-employed, including unemployed and retired, homemakers, and students), smoking and alcohol use, anthropometric measures (weight and height), and laboratory results.

### Weight and height measurements

We ensured that all adults with T2DM wore light clothing and went barefoot during weight and height measurements. The weight was measured in kilograms using a scale, which was zeroed before each measurement to the nearest 100 g. Adults were asked to stand upright, back against the wall, and feet together, and to have their height measured to the nearest 0.1 cm using a Seca stadiometer. BMI was computed by dividing participants’ weight in kilograms (kg) by their height in square meters (m^2^). BMI was grouped based on the “World Health Organization (WHO) classification into underweight, normal weight, overweight, or obese (15)”.

Various tools are used to assess cognitive status in different populations, including the Montreal Cognitive Assessment (MoCA) and Mini-Mental State Examination (MMSE) (16, 17). The MoCA is widely used worldwide as a screening tool for cognitive evaluation in surveys and has higher sensitivity than the MMSE for detecting cognitive impairment (17, 18).

### Cognitive health assessment

The Arabic version of the MoCA was used in this study to evaluate cognition, including executive function, memory, language, attention, visuospatial skills, and orientation. The Arabic version of the MoCA has excellent accuracy, with a test-retest correlation (r = 0.92), Cronbach’s alpha = 0.83, and sensitivity and specificity for mild cognitive impairment detection of 92.3% and 85.7%, respectively (19). The MoCA has a total score that ranges from 0 to 30 (17). Then the MoCA scores are categorized into four levels: normal (26–30), mild (19–25), moderate (11–18), and severe (0–10). We adjusted MOCA score cutoffs for education by adding a point for adults with ≤12 years of schooling. The MoCA has been previously used in Sudan (20). All predictor data and MoCA assessments were collected during the October-November 2025 study period.

### Dependent variable

#### Cognitive impairment

##### Independent variables

Sociodemographic variables (age, sex, education, marital status, residence, occupation), smoking and alcohol use, BMI, duration of DM, diabetes control, and hyperlipidemia were the confounders that have been reported to be associated with cognitive impairment in patients with DM in previous studies (4, 5, 7, 8).

### Operational definitions

In this study, T2DM was diagnosed in adults according to the American Diabetes Association diagnostic criteria (21). Controlled T2DM was considered when hemoglobin A1c was<7.0%. Hyperlipidemia was diagnosed if one or all of the following criteria were fulfilled: total cholesterol > 200 mg/dl, LDL > 100 mg/dl, HDL< 40 mg/dl, triglyceride > 150 mg/dl (22).

### Data analysis

Data analysis was performed using SPSS version 25. The Shapiro–Wilk test was used to assess the normality of all continuous data (age, duration of DM, BMI, and hemoglobin A1c), which were non-normally distributed and reported as medians (interquartile ranges). Nonparametric (Mann–Whitney U) and chi-square tests were used to compare the medians and proportions between the two groups (with and without cognitive impairment), respectively. Univariate logistic regression was performed, and variables with P ≤ 0.2 were included in a multivariate logistic regression (backward Likelihood Ratio elimination) to identify factors significantly associated with cognitive impairment. Collinearity (variance inflation factor > 4) was checked for and did not exist.

## Results

### General characteristics

Of 400 patients with T2DM, 276 (69.0%) were females, and 361 (90.3%) were married. The median (interquartile range, IQR) of age, duration of T2DM, and BMI was 53.0 (45.0 – 61.0) years, 7.0 (2.1–12.0) years, and 25.5(22.7–28.9) kg/m^2^. Of the 400 patients, 257 (64.2%) were urban residents, and 266 (66.5%) were non-employees. A significant proportion of the patients (73.0%) had an education level below secondary school. Fifty-nine (14.8%) patients were current or former smokers, and 30 (7.5%) were current or former alcohol consumers. Of 400 patients, 159 (39.8%) had a normal weight, 18 (4.5%) were underweight, 148 (37.0%) were overweight, and 75 (18.8%) were obese. Of 400, 322(80.5%) had uncontrolled T2DM. Eighty-seven (21.7%) patients had hyperlipidemia, and 111(27.7%) were hypertensive (**Table 1**).

**Table 1:** Characteristics of patients with type 2 diabetes mellitus in Gadarif, eastern Sudan (number =400), 2025.

Variable			
		Median	Interquartile range
Age, years		53.0	45.0 – 61.0
Duration of diabetes mellitus, years		7.0	2.1–12.0
Body mass index, kg/m^2^		25.5	22.7–28.9
Hemoglobin A1c, %		9.2	7.3–11.4

Category		Number	Percentage
Sex	Female	276	69.0
	Male	124	31.0
Marital status	Married	361	90.3
	Unmarried	39	9.7
Residency	Urban	257	64.2
	Rural	143	35.8
Employment Status	Employee	134	33.5
	Non-employee	266	66.5
Education level	Secondary and above	108	27.0
	Less than secondary	292	73.0
Smoking status	Never	341	85.2
	Current or former	59	14.8
Alcohol status	Never	370	92.5
	Current or former	30	7.5
Body mass index	Normal	159	39.8
	Underweight	18	4.5
	Overweight	148	37.0
	Obese	75	18.8
Hypertension	No	289	72.3
	Yes	111	27.7
Uncontrolled type 2 Diabetes mellitus	No	78	19.5
	Yes	322	80.5
Hyperlipidemia	No	313	78.3
	Yes	87	21.7

### Cognitive impairment

The MOCA score ranged from 7 to 30 points with a median of 22.0 points. When the MOCA score was divided, the level was normal in 94 (23.3%), mild in 170 (42.2%), moderate in 125 (31.3%), and severe in 11(2.8%) patients. Three hundred and six (76.5%) patients with T2DM had cognitive impairment.

### Factors associated with cognitive impairment

The median (IQR) age [54.0 (45.0–63.0) years versus 50.0 (44.7–58.2) years; P = 0.010], BMI [25.6 (22.7–29.3) kg/m^2^ versus 25.2 (22.4 –28.1) kg/m^2^, P = 0.002] and duration of T2DM [7.0 (3.0–12.0) years versus 5.0 (1.0–11.2) years, P = 0.044] was significantly higher in patients with cognitive impairment than that of those without cognitive impairment. Significantly higher numbers of patients with cognitive impairment were females, non-employees, had a lower level of education, and were alcohol consumers, compared with patients without cognitive impairment. There was no difference in marital status, residency, smoking status, and hyperlipidemia between patients with and without cognitive impairment (**Table 2**).

**Table 2:** Comparing the number (percentage) of characteristics of patients with type 2 diabetes mellitus with and without cognitive impairment in Gadarif, eastern Sudan, 2025.

Variables		Patients with cognitive impairment (number 306)	Patients without cognitive impairment (number= 94)	P value
	Median (interquartile range) of			
Age, years		54.0 (45.0–63.0)	50.0 (44.7–58.2)	0.010
Body mass index, kg/m^2^		25.6 (22.7–29.3)	25.2 (22.4 –28.1)	0.002
Duration of diabetes, years		7.0 (3.0–12.0)	5.0 (1.0–11.2)	0.044
Hemoglobin A1c%		9.2 (7.4–11.5)	8.8 (7.1–11.0)	0.453
	Number (percentage) of			
Sex	Female	222 (72.5)	54 (57.4)	0.007
	Male	84 (27.5)	40 (42.6)	
Marital status	Married	278 (90.8)	83 (88.3)	0.435
	Unmarried	28 (9.2)	11 (11.7)	
Residency	Urban	191 (62.4)	66 (70.2)	0.178
	Rural	115 (37.6)	28 (29.8)	
Employment status	Employee	78 (25.5)	56 (59.6)	<0.001
	Non-employee	228 (74.5)	38 (40.4)	
Education level	≥Secondary	44 (14.4)	64 (68.1)	<0.001
	< Secondary	262 (85.6)	30 (31.9)	
Smoking status	Never	257 (84.0)	84 (89.4)	0.245
	Current/former	49 (16.0)	10 (10.6)	
Alcohol status	Never	277 (90.5)	93 (98.9)	0.003
	Current/former	29 (9.5)	1(1.1)	
Uncontrolled type 2 diabetes mellitus	No	58 (19.0)	20 (21.3)	0.656
	Yes	248 (81.0)	74 (78.7)	
Hyperlipidemia	No	237 (77.5)	76 (80.9)	0.568
	Yes	69 (22.5)	18 (19.1)	

In univariate logistic regression, age (OR = 1.02, 95.0% CI = 1.01–1.04), female sex (OR = 1.95, 95.0% CI = 1.21–3.16), lower level of education (OR = 12.70, 95.0% CI = 7.41–21.76), non-employee (OR = 4.30, 95.0% CI = 2.65–7.00), and alcohol status (OR = 9.73, 95.0% CI = 1.30–72.46) were associated with cognitive impairment. Duration of T2DM, BMI, marital status, residency, education level, smoking status, hypertension status, hemoglobin A1c/poor control of T2DM, and hyperlipidemia were not associated with cognitive impairment (**Table 3**).

**Table 3:** Univariate and multivariate binary analysis of factors associated with cognitive impairment among patients with type 2 diabetes mellitus in Gadarif, eastern Sudan, 2025.

Variables		Univariate binary analysis		Multivariate binary analysis	
		Non-adjusted odds ratio (95% confidence interval)	P value	Adjusted odds ratio (95% confidence interval)	P value
Age, years		1.02 (1.01–1.04)	0.014	1.01 (0.98–1.03)	0.688
Body mass index, kg/m^2^		1.01 (0.96–1.06)	0.608		
Duration of diabetes, years		1.02 (0.98–1.06)	0.196	1.02 (0.98–1.07)	0.195
Hemoglobin A1c		1.03 (0.95–1.13)	0.404		
Sex	Male	References		References	
	Female	1.95 (1.21–3.16)	0.006	0.55 (0.27–1.22)	0.100
Marital status	Married	References			
	Unmarried	0.76 (0.36–1.59)	0.467		
Residency	Urban	References		References	
	Rural	0.70 (0.42–1.16)	0.169	0.87 (0.47 –1.62)	0.679
Employment status	Employee	References			
	Non-employee	4.30 (2.65–7.00)	<0.001	2.23 (1.16–4.29)	0.016
Education level	Secondary and above	References			
	Less than secondary	12.70 (7.41–21.76)	<0.001	9.93 (5.51–17.90)	<0.001
Smoking status	Never	References	0.202		
	Current or former	1.60 (0.77–3.30)			
Alcohol status	Never	References	0.026	References	
	Current or former	9.73 (1.30–72.46)		8.21 (1.02–66.02)	0.048
Hypertension	No	Reference			
	Yes	1.56 (0.90–2.71)	0.211		
Hyperlipidemia	No	References			
	Yes	1.22 (0.68–2.19)	0.485		
Uncontrolled type 2 diabetes mellitus	No	References			
	Yes	1.15 (0.65–2.04)	0.619		

Multivariate logistic regression showed that lower levels of education (OR = 9.93, 95.0% CI = 5.51–17.90), non-employee status (OR = 2.23, 95.0% CI = 1.16–4.29), and alcohol status (OR = 8.21, 95.0% CI = 1.02–66.02) were associated with cognitive impairment (**Table 3**). The model’s goodness of fit was assessed using the Hosmer-Lemeshow test, which indicated a good fit (*P =* 0.923).

## Discussion

The main findings of the current study were that 76.5% of the adults with DM had cognitive impairment, which was associated with lower levels of education, non-employee status, and alcohol status. This rate of cognitive impairment was similar or slightly lower than that reported in a community-based survey in central Sudan, which showed that 73.5% of the adults had cognitive impairment (MoCA score< 26) (12). The prevalence of cognitive impairment in this study was significantly higher than that reported in Amhara National Regional State referral hospitals, Ethiopia (46.27%) (4) and in the Northwest, Ethiopia, 28.3% (5), Uganda ((MoCA) (7),, and in Congo, 18.7% (9) in patients with DM. In a systematic review, the pooled prevalence of cognitive impairment in patients with DM was 43.9% (6). However, the rate of cognitive impairment in the current study was lower than that reported in Nigeria (88.5%) (8).

Educational profile, healthcare disruption due to the Sudan conflict, selection of tertiary-center patients, MoCA cutoff choice, and referral bias could explain the differences between our results and those in other populations.

In the current study, lower education levels were associated with cognitive impairment. This is in agreement with several previous studies in African countries, which showed that a low level of education was associated with cognitive impairment (4, 6, 7). For example, in a community-based survey in Sudan, multivariate binary analysis showed that factors independently associated with cognitive impairment were low education level, unemployment, and depression) (12). In the Amhara National Regional State referral hospitals, Ethiopia, no formal education was associated with cognitive impairment (4). However, in our results, nearly 73% of participants had less than secondary education. Low educational attainment is known to substantially influence MoCA performance. The observed prevalence of 76.5% may therefore reflect educational bias rather than true cognitive impairment.

In this study, alcohol was associated with cognitive impairment. However, our results showed a wide range of CIs (1.02–66.02), which may be due to small subgroup sizes and should be interpreted with caution. Alcohol is a male habit in Sudan, and even males might not disclose their history of alcohol use easily, as they feel ashamed. Several previous studies have shown that alcohol was associated with cognitive impairment (4, 5, 7). For example, alcohol was associated with cognitive impairment in Amhara National Regional State referral hospitals, Ethiopia (4), in the Northwest, Ethiopia (5), and in Uganda (7). Several changes, such as hyaline arteriolosclerosis, neurofibrillary tangles, and a lower brain mass ratio, were associated with alcohol consumption (23). The use of alcohol may lead to structural as well as functional brain damage, leading to what is known as alcohol-related dementia (24).

Our results showed that adults who were not employed were at higher risk for cognitive impairment. This is in agreement with a previous study in Sudan that showed unemployment was associated with cognitive impairment (12).

In this study, sex and duration of DM were not associated with cognitive impairment. In the Northwest of Ethiopia, females are at greater risk of cognitive impairment (5). Previous studies in Amhara National Regional State referral hospitals, Ethiopia (4) and in the Northwest, Ethiopia, 28.3% (5) in Uganda (7), in Congo (9) and in a systematic review, patients with a longer duration of DM have congestive impairment (6).

Our results have shown no association between marital status, residency, smoking status, hypertension, hyperlipidemia, BMI, hemoglobin A1c, and cognitive impairment. Several previous studies have shown that these factors, such as older age (4, 6, 8, 9), rural residence (5), obesity (4, 8), co-morbidity (5, 7), and hyperglycemic episodes (4), have been reported to be associated with cognitive impairment in patients with DM in different African countries (4).

Our results differ from those reported in other African countries in that sociodemographic characteristics such as age, sex, marital status, residency, and smoking status, as well as metabolic factors such as hypertension, hyperlipidemia, BMI, and hemoglobin A1c/poor control of T2DM, were not associated with cognitive impairment. The difference between our results and those of others could be explained by differences in socioeconomic characteristics, age of enrolled patients, and the tools used to assess cognitive impairment.

## Limitation

This was a single-center study, and its results may not generalize. It was a cross-sectional study, which does not allow causal inference. Moreover, alcohol history was self-reported, and there was possible educational bias in MoCA. Although one point was added for participants with ≤12 years of education, MoCA performance may still be influenced by education. MoCA is a screening tool for mild cognitive impairment but may miss early cognitive decline and cannot reliably distinguish mild cognitive impairment from dementia syndromes. Selecting potential variables based on previous studies does not demonstrate a prespecified approach for this study. Each variable should be selected in advance with a clear rationale. Using a statistical method simply because previous studies used it may not be appropriate for every study. Several established determinants of cognitive impairment, such as depression, anxiety, medication adherence, history of stroke, chronic kidney disease, retinopathy, neuropathy, hypoglycemic episodes, physical activity, and socioeconomic status, were not measured.

## Conclusion

Three out of four patients with T2DM had cognitive impairment in East Sudan. Low level of education, non-employee status, and alcohol consumption are associated factors. There is a need for public health interventions so as to detect and manage cognitive impairment early in adults with T2DM.

## Data Availability

The raw data supporting the conclusions of this article will be made available by the authors, without undue reservation.
